# Impact of Histone Lysine Methyltransferase SUV4-20H2 on Cancer Onset and Progression with Therapeutic Potential

**DOI:** 10.3390/ijms25052498

**Published:** 2024-02-21

**Authors:** Stela Papadaki, Christina Piperi

**Affiliations:** Department of Biological Chemistry, Medical School, National and Kapodistrian University of Athens, 75 M. Asias Street, 11527 Athens, Greece; stylianipapadaki@outlook.com.gr

**Keywords:** lysine methyltransferase, epigenome, epigenetic modifications, chromatin compaction, heterochromatin, gene regulation, histone modifications, DNA methylation, cancer

## Abstract

Histone lysine methyltransferase SUV4-20H2, a member of the suppressor of variegation 4–20 homolog (SUV4-20) family, has a critical impact on the regulation of chromatin structure and gene expression. This methyltransferase establishes the trimethylation of histone H4 lysine 20 (H4K20me3), a repressive histone mark that affects several cellular processes. Deregulated SUV4-20H2 activity has been associated with altered chromatin dynamics, leading to the misregulation of key genes involved in cell cycle control, apoptosis and DNA repair. Emerging research evidence indicates that SUV4-20H2 acts as a potential epigenetic modifier, contributing to the development and progression of several malignancies, including breast, colon and lung cancer, as well as renal, hepatocellular and pancreatic cancer. Understanding the molecular mechanisms that underlie SUV4-20H2-mediated effects on chromatin structure and gene expression may provide valuable insights into novel therapeutic strategies for targeting epigenetic alterations in cancer. Herein, we discuss structural and functional aspects of SUV4-20H2 in cancer onset, progression and prognosis, along with current targeting options.

## 1. Introduction

Cancer onset involves the deregulation of developmental and homeostatic pathways, resulting in the acquisition of certain cellular characteristics, interacting systemically to induce pathogenesis. Such traits include uncontrolled cellular proliferation, nonoxidative metabolism, metastasis and invasiveness, genomic instability, tumor-induced immune activation and resistance to growth suppressor stimuli, acquired through differential gene regulation [1]. This process can be achieved either directly, implicating transcriptional factors downstream of signaling molecules, or indirectly, through a complex network of biochemical modifications/marks established on gene bodies or regulatory areas. This set of alterations is termed the epigenome and refers to a number of reversible modifications that finetune gene expression in accordance with stimuli and time [2]. These modifications include DNA methylation, covalent and noncovalent histone modifications and the repositioning of nucleosomes and noncoding RNAs that participate in chromatin structure [3]. It is important to highlight that these alterations do not alter the DNA sequence despite the fact that some of them use it as a binding scaffold. Moreover, these alterations have different impacts on gene expression regulation, with some of them activating and others suppressing gene transcription.

Cancer cells are characterized by gene deregulation often associated with epigenetic marks that suppress transcription, which are localized on tumor-suppressive genes, and interfere with cell cycle regulation [2]. On the other hand, epigenetic alterations that activate transcription can be established on gene promoters, creating a permissive state that allows oncogenes’ expression. Additionally, imprinted genes, which are reported to be regulated through epigenetic silencing in normal physiology, have been detected to bear a significant loss of suppressive marks, with the illustrative example of insulin-like growth factor 2 (*IGF2*), which is upregulated in a vast majority of cancers [4]. Central processes such as invasion and metastasis, characterized by a transient genetic profile, are also highly regulated through epigenetic marks [5]. The reversible nature of epigenetic alterations renders them an appealing target for intervention and drug development, with a range of different strategies aiming at writers (enzymes that establish epigenetic marks), erasers (enzymes that remove the marks) or even readers (enzymes that bind the marks and allow other molecular interactions to take place) [6,7].

### 1.1. DNA Methylation: A Key Epigenetic Mechanism in Cancer

Among the main epigenetic mechanisms, DNA methylation has been extensively studied and linked to cancer initiation and development, taking place at the 5′ ends of genes on cytosine residues of CpG dinucleotides in 60% of human genes [3,8]. DNA methylation is a central regulatory mechanism for gene expression during development, with CpG methylation levels varying according to the stage of the process, thus regulating genes necessary for the respective developmental stages [9,10]. High levels of DNA methylation are observed in repetitive genomic sequences, where heavy methylation attenuates genome instability. Examples of such genomic areas include transposable elements, noncoding DNA sequences and long interspersed nuclear elements [11]. In this background, DNA methylation serves as a stabilizing agent that protects chromatin structure and integrity. It is worth noting that DNA methylation can serve as a docking site for proteins while stabilizing chromatin structure. As an epigenetic mark present in gene promoters, DNA methylation serves as a repressor of transcription with a very important contribution not only in the correct regulation of development but also in the establishment of X-chromosome inactivation and repression of imprinted genes [12]. A reported mechanism that explains the repressive effect of DNA methylation, in some contexts and with regard to some transcription factors, involves the recruitment of proteins capable of recognizing CpG-methylated dinucleotides. These proteins also have the ability to simultaneously recruit histone deacetylases (HDACs), enzymes that remove acetylation marks from histones, thus creating a transcriptional repressive environment [13,14,15,16]. The establishment of DNA methylation takes place through three DNA methyltransferases (DNMTs), whereas removal of the mark is accomplished through the ten-eleven translocation family of dioxygenases (TET enzymes). 

In cancer, DNA methylation follows different patterns in distinct genomic regions. For example, in CG-rich promoters, DNA methylation can increase dramatically, thus repressing the expression of genes such as tumor suppressor or DNA repair- and cell cycle-related ones [8]. As a result, cell proliferation is left unchecked, either directly or indirectly. Examples include the breast cancer gene 1 (*BRCA1*) in ovarian and breast cancer, the Von Hippel–Lindau tumor suppressor (*VHL*) in clear renal cell carcinoma and the O-6-methylguanine-DNA methyltransferase (*MGMT*) repair gene in glioblastoma (GB) [17]. Hypomethylation, on the other hand, has been reported on imprinted genes such as *IGF2*/*H19*, which is responsible for the production of epidermal growth factor 2 (EGF2), in intragenic and intergenic regions where long or short interspersed elements can be found. Consequently, differential production of regulating factors and high motility of retrotransposons are observed, leading to increased proliferating signals and chromosome instability, respectively [18].

### 1.2. The Role of Histone Modifications in Cancer

DNA methylation is not the only epigenetic mechanism with a prominent role in carcinogenesis. Histone modifications have also been detected in all cancer types, established post-translationally and exerting pivotal roles in gene expression regulation and chromatin structure. Histone modifications encompass a variety of chemical changes in N-terminal amino acids of histone proteins including methylation, acetylation, SUMOylation, ADP-ribosylation and ubiquitination [3]. Among them, histone methylation plays a prominent role in the activation as well as the suppression of gene transcription depending on the modified amino acid in the respective histone member.

Methylation of histones is a well-studied mark, context-dependent and tightly associated with the aforementioned processes. It can take place on lysine or arginine residues of H3 and H4 in different states or degrees of methylation, with each level bearing distinct regulatory effects in normal physiology [19]. The establishment of histone methylation on lysine residues takes place through the enzymatic activity of six protein families, each one of them targeting different residues on histone molecules [20]. The same number of families exist also for the reverse reaction, without the protein family distinction representing substrate specificity. Lysine methylation can be grouped into activating signals such as H3K4, H3K36, H3K79 and repressive marks such as H3K9 and H4K20 [20]. 

Deregulation of enzymes involved in the addition or removal of methyl group(s) from lysine or arginine residues has been involved in cancer [21]. Arginine histone methylation is mediated through the respective group of enzymes named arginine methyltransferases (PRMTs), which can induce mono- or dimethylation of histones in a symmetrical and nonsymmetrical way [22]. The removal of arginine methylation marks takes place through deamination, yielding citrulline as an end product. In hematological and solid tumors, the levels of PRTMs appear to be significantly elevated and correlate with poor patient survival [23]. An intriguing fact about PRMTs is their ability to create both activating and repressive marks by cooperating with distinct proteins on different promoters. In the case of acute myeloid leukemia, H4R3me2a participates in epithelial–mesenchymal transition (EMT) by regulating zinc finger E-box binding homeobox 1 (*ZEB1*) promoter, while its demethylation under the influence of specific factors is implicated in leukemic transformation.

Regarding histone lysine methylation, more data and genome-wide analyses are present that indicate a pivotal role in carcinogenesis. In breast cancer, a set of KMTs (histone lysine methyltransferases) was associated both on the mRNA and protein level with poor prognosis, bearing alterations in gene sequence as well as in expression levels and, thus, transforming the methylation levels of their respective histone lysine targets [24]. It is of note that in different types of breast cancer or in different stages of the disease, a distinct set of these enzymes is aberrantly expressed, demonstrating yet again the contextual nature of these marks [25]. In gastric cancers, high levels of enhancer of zeste 2 polycomb repressive complex 2 subunit (EZH2), a writer of H3K27me3, results in gene repression, whereas the epigenetic mark levels have been reduced when the core components of the polycomb complex are reduced, indicating the contextual nature of epigenetic marks in the heterogenous cancer background [26]. Additional examples of deregulated activity of histone methylation writers and erasers have been observed in digestive cancers [27]. 

In this review, we are particularly interested in the role of a relatively obscure histone lysine methyltransferase that has gained attention recently in several cancer types, named suppressor of variegation 4-20 homolog 2 (SUV4-20H2). We discuss the structural and functional characteristics of this enzyme in normal physiology and its involvement in cancer onset and progression, aiming to identify specific targeting options for future studies.

## 2. Biochemical Aspects of SUV4-20H2

SUV4-20H2 is a protein coded by the gene *KMT5C* in the human genome. The transcription of the gene generates two isoforms; one consists of 462 amino acids, which corresponds to the canonical sequence and lays under the UniProt database (code Q86Y97-1), whereas the second transcriptional product has only 97 amino acids, of which only the first 36 are identical between the two isoforms. According to the database, the second product is expected to be found only in very low levels due to the existence of a premature stop codon in the mRNA. However, in this review, we describe the structural and functional properties of the isoform corresponding to the canonical sequence [28]. 

### 2.1. SUV4-20H2 Structural Characteristics

SUV4-20H2 is a lysine methyltransferase member of the suppressor of variegation 4-20 homolog (SUV4-20) family that bears a catalytic SET domain responsible for the methylation of the substrate, an N-terminal domain, an inserted motif that divides the SET domain into two parts and a post-SET domain located at the C-terminal [29]. Among these parts, only two amino acid residues bear post-translational modifications according to the UniProt database, namely Thr416 and Thr422, which appear to bear phosphorylation marks, while two more are suspected to occur further down the C-terminal end, based on large data collected through bioinformatic analysis. The SET domain of SUV4-20H2 appears to be relatively distinct from the typical SET domain, while at the same time, it maintains some of its typical core features. For example, the canonical β-sheet topology is reported for SUV4-20H2 [29], while the amino acid sequence bears little to no similarity with other SET domain-containing proteins [30], highlighting the importance of structural integrity for the methylation of the targeted lysine residues. 

**Figure 1:**
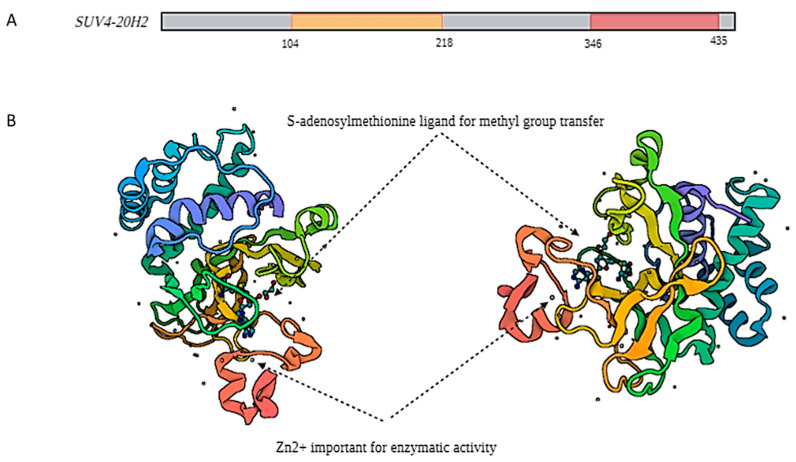
The structure of SUV4-20H2 (*KMT5C* gene) in a linear domain scheme (**A**) and in ribbon scheme (**B**), as deposited in PDB database. The green/orange area of the structure corresponds to the SET domain (104–218 aa) with the characteristic β sheets and the heterochromatin binding domain (346–435 aa). The domain is in close proximity to the active site of the methyltransferase and contains important binding sites for SAM (S-adenosylmethionine), EDO (ethanodiol) and histone H4 area depicted in red. The left side of the picture illustrates the protein sideways, allowing a better observation of the location of Zn^2+^, whereas the right side represents a frontal representation. The Biorender software was used for the illustration and the structure was imported directly from PDB [33].

The typical Zn binding motif is localized right after the SET domain appears distinct between SUV4-20H2 and other SET proteins [31]. The four cysteine residue motif that is normally present on SET proteins is replaced by a pattern of three cysteines with a different structure. The classical pattern is CxCxxxxC inside the post-SET domain, where x corresponds to any other amino acid [32] and an extra amino acid inside the SET domain (Figure 1). 

In SUV4-20H2, the three cysteine residues form an α-helix, and all of them are found within the post-SET domain with a sequence of CxCxxC [30]. It is worth pointing out that the enzymatic activity of SET domain-containing proteins is in tight association with the surrounding domains and plays a pivotal role in defining substrate specificity. Another feature that distinguishes SUV4-20H2 is the presence of only five α-helices in its N-terminal end, as opposed to SUV4-20H1, which bears six (Figure 1) [30].

### 2.2. SUV4-20H2 Enzymatic Properties

Southall et al. [30] have extensively investigated the enzymatic properties of SUV4-20H2 and calculated a slightly lower Km for a monomethylated synthetic peptide of H4K20, consisting of the 24 first amino acid residues of the H4 histone as opposed to the unmethylated version of the synthetic peptide, indicating a preference for the monomethylated substrate (16 ± 2.8 μΜ vs. 21 ± 2 μΜ). Under the same conditions, the turnover rate (Kcat) is approximately three times higher for the monomethylated substrate as opposed to the unmethylated one. It is of note that when the enzyme was tested against the nucleosome, Km dropped significantly (0.5 ± 0.1 μΜ) while Kcat increased, leading to a final ratio of 10.6 μM^−1^ h^−1^ and thus demonstrating the preference of the enzyme for the naturally occurring conformation of the histone. For the dimethylated peptide, no activity was detected under these experimental conditions, suggesting that at least in vitro the preferential substrate is the monomethylated H4K20. The study showed that the aromatic pocket of the SET domain, which is the active site for methylation reaction, contains a serine residue, which aligns the targeted lysine for mono- or dimethylation but not trimethylation and, thus, controls the methylation status of the product. 

Another investigation [34] on the enzymatic activity of both enzymes corroborated the high preference of SUV4-20H2 for the monomethylated H4K20 substrate while at the same time demonstrating the production of H4K20me2. The study suggested that the absence of H4K20me3 in both experimental procedures might be a result of the applied conditions. At the same time, the presence of S161 in the amino acid sequence was indicated as a possible explanation for the inability of the enzyme to provide a product of higher methylation status, given that the hydrogen bond between the serine residue in question and the targeted lysine impedes the binding of a second methyl group, thus allowing for the detection of only demethylated products. Comparative analysis among differentially generated peptides and the two enzymes showed that SUV4-20H2 has a similar recognition motif as SUV4-20H1 but is less specific with its respective sequence. The lower specificity of the motif sequence suggested that apart from histone 4, other proteins may also serve as methylation targets for SUV4-20H2 based on the presence of the aforementioned motif in their amino acid sequence, identifying Opa interacting protein 5 (OIP5) and Centromere protein U (CENPU) as possible targets [34]. It is important to note that these in vitro experiments were conducted with a bacterially produced N-terminal part of the enzyme, and possible post-translational modifications as well as the importance of the integrity of the 3D structure might have been overlooked.

## 3. SUV4-20H2 Functions in Normal Physiology

**Figure 2:**
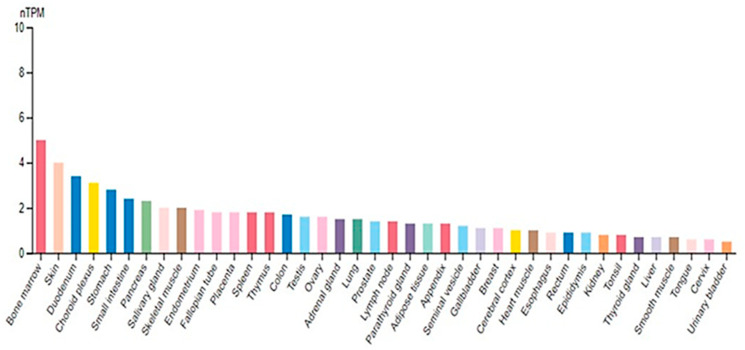
SUV4-20H2 expression levels in normal tissue types. The diagram shows RNA-seq expression levels (normalized) for the HPA RNA-seq tissue data reported as nTPM (normalized protein-coding transcripts per million), corresponding to mean values of the different individual samples from each tissue. Color-coding is based on tissue groups, each consisting of tissues with functional features in common (https://www.proteinatlas.org/ENSG00000133247-KMT5C/tissue#rna_expression, accessed on 22 January 2024).

SUV4-20H2 is ubiquitously expressed in almost all tissues, presenting the highest levels at the G1 and S phases of the cell cycle (Figure 2). Aberrant regulation of SUV4-20H2 has been associated with impaired cell proliferation and elevated sensitivity to DNA damage [35]. SUV4-20H2 exhibits a high affinity for H4K20me1 and uses it as a substrate to establish the important repressive mark, H4K20me3. H4K20 methylation marks (H4K20me2/3) have been demonstrated to be essential for normal development since SUV4-20H double-knockout mice are perinatally lethal and exhibit loss of H4K20 marks, associated with defective DNA repair and elevated genomic stress [36]. 

Based on its activity as a writer, SUV4-20H2 exhibits multiple roles in physiology, being involved in key biological processes such as DNA replication, recombination and maintenance of proper telomere length, as well as transcriptional regulation through the establishment of heterochromatin and RNA polymerase II (Pol II) pausing [31]. Given the imperative role of these processes for cellular homeostasis, deregulation, depletion or even knockdown of *KMT5C* has been involved in altered and pathologic phenotypes, including embryonic developmental defects, differentiation of embryonic stem (ES) cell regulation, maturation and class switch recombination in B cells, maturation of erythrocytes and neuroectodermal differentiation.

### 3.1. SUV4-20H2′s Role in DNA Replication

The implication of SUV4-20H2 in the DNA replication of somatic cells is attributed to its methyltransferase role since H4K20 methylation states are essential for proper DNA replication. SUV4-20H1/2 have been shown to enable PR-Set7 methyltransferase to recruit the origin recognition complex (ORC) to chromatin [35]. Moreover, the H4K20me3 repressive mark is involved in timely replication during the S phase, assisting the correct replication timing at specific heterochromatin regions [37]. During embryonic development, the H4K20 methylation status is tightly regulated, with H4K20me1 being the only mark present after fertilization and at pre-implantation stages, while H4K20me3 accumulates slowly during fetal development [38]. To this end, the absence of H4K20me3 is associated with high developmental potency and ectopic SUV4-20H2 expression at this stage, resulting in developmental defects [38]. Additionally, SUV4-20H2 has been involved in embryonic stem (ES) cell differentiation and repression of lineage-specific genes in ES cells [39].

### 3.2. SUV4-20H2 Implication in B Cell and Erythrocyte Maturation

Another important function of SUV4-20H2 is its implication in the immunoglobulin class switch recombination (CSR) process involved in the maturation of B cells, a central process for the development of innate immunity in higher organisms. The process takes place in primary lymphoid organs and allows for the production of a great variety of antibodies with each type being activated under different stimuli [40]. CSR, followed by somatic hypermutations, is mediated by the activation-induced cytidine deaminase (AID) enzyme, which has the ability to switch SUV4-20H1 to SUV4-20H2 in Smu sites of the IgH locus [40]. Furthermore, it was demonstrated that B cells from AID-deficient mice had reduced levels of H4K20me3 in these sites during class switch recombination, whereas cells from normal mice activated for CSR with lipopolysaccharide (LPS) and IL-4 had the exact opposite phenotype. A possible explanation for this trait involves the promoter proximal pausing of RNA pol II, which has been shown to allow Pol II to bind through St5 on the targeted sequences, thus allowing other histone modifiers to alter epigenetic marks and change the transcriptional environment to a permissive state needed for the production of the antibodies [40]. The study of Kapoor-Vazirani et al. demonstrated that RNA pol II promoter proximal pausing is a limiting step in the transcription of a variety of genes, regulated by the balance of H4K16Ac and H4K20me3 [41]. In greater detail, it was observed that CpG hypermethylation and epigenetic silencing of *TMS1* (target of methylation-induced silencing 1) was followed by an increase in H4K20me3, while the levels of H4K16Ac were reduced. At the same time, it was shown that 20–30% of the genes in the human genome are on pause with a small transcript being produced, vastly smaller in length than the canonical sequence, indicating the possibility that the initiation of elongation during transcription is a rate-limiting step. During their study, it became evident that lysine acetyltransferase 8 (hMOF) the enzyme responsible for H4K16Ac, allows for the release of the paused RNA Pol II enzyme, whereas SUV4-20H2 antagonizes this effect through the establishment of H4K20me3. However, in the absence of this particular epigenetic mark, no such pausing was observed, indicating a possible gene-specific mechanism, observed only on genes bearing this particular epigenetic mark where the interplay between H4K16Ac and H4K20me3 takes place. It is of note that other epigenetic gene silencing signals present, such as DNA methylation or H3K9me2, do not have the same effect on Pol II pausing, highlighting the complexity of transcriptional regulation. 

Another biological process that is based on this particular ability of SUV4-20H2 to regulate transcription is the maturation of erythrocytes. Erythropoiesis results in the production of erythroid progenitor cells with specific characteristics, which include the high degree of compaction of chromatin and the reduced production of RNA that allow for the maturation of progenitor cells to enucleated erythrocytes. The study of Gillinger et al. showed that SUV4-20H2 is a downstream target of pSTAT5 through an intronic enhancer of the gene [42]. This interaction is thought to enhance SUV4-20H2 production in murine cells, thus increasing the cellular levels of H4K20me3, which in turn induces transcriptional pause, as described above. Consequently, genes related to erythropoiesis are gradually silenced and chromatin is condensed, two steps necessary for the enucleation that is crucial for the release of mature erythrocytes in blood circulation.

### 3.3. SUV4-20H2 Implication in Heterochromatin Formation and Chromosomal Integrity

Furthermore, SUV4-20H2 regulates telomere length homeostasis through H4K20 methylation [43]. Telomere length maintenance is accomplished by restricting access to telomerase through the establishment of heterochromatin marks, such as H3K9me3, H4K20me3 and high levels of DNA methylation. Cells deficient to SUV4-20H1/H2 enzymes, either simultaneously or interchangeably, showed decreased H4K20me3 levels at telomeres and subtelomeres and increased telomere length as well as increased telomere recombination, while all other epigenetic marks remained the same. These mechanistic experiments took place in MEFs and ES cells and highlighted yet again the specificity of SUV4-20H2 as the main enzyme that produces the trimethylated mark, given that upon SUV4-20H1 knockdown, no alterations were observed [43]. 

The most well-recognized role of SUV4-20H2 and H4K20me3 is the maintenance of constitutive heterochromatin and its impact on gene transcription and expression. The pericentric heterochromatin (PCH) bears two epigenetic marks of crucial importance: H3K9me3 established by SUV39H1/2 and H4K20me3 mediated by SUV4-20H2. These epigenetic signatures are sequentially established given that H3K9me3 acts as a binding scaffold not only for heterochromatin protein 1 (HP1) but also for SUV39H1/2, thus creating a positive feedback loop. HP1 proteins bear a CSD domain that allows for their dimerization as well as the binding of other factors related to heterochromatin, such as SUV4-20H2, which can further methylate lysine residues in H4 histone [44]. H4K20me3 is related to cohesin enrichment and plays a central role in maintaining proper chromosome segregation through the cell cycle. Cohesin is a complex of dimers that forms a ring structure that can be loaded on DNA in the G1 phase of the cell cycle and removed during anaphase by proteolytic degradation, enabling proper separation of sister chromatids [45]. It is of note that the ring structure is not immobile but can slide along through the action of CTCF (CCCTC binding factor, zinc finger protein), a versatile protein with the ability to bind DNA and regulate not only transcription but also the formation of DNA loops. Initial experiments on ES cells and fibroblasts demonstrated that SUV4-20H2 can interact with HP1 proteins through a number of interaction sites in its C-terminal domain, inducing a more compacted form of chromatin. In vitro experiments highlighted the importance of the C-terminal domain for the proper establishment of heterochromatin and also demonstrated that the full length of the protein is important for intrachromosomal interactions [46]. Cells lacking SUV4-20H2 exhibited improper chromosome segregation and reduced sister chromatid cohesion, while cells expressing mutated forms of the enzyme showed lower chromatin compaction, altered chromocenter organization in interphase and a substantial decrease in cohesion levels [46].

In vitro experiments conducted in mouse embryonic fibroblasts (MEFs) derived from knockout (KO) mice for the HP1 protein family genes demonstrated that HP1β has a higher affinity for H4K20me3 than the other isoforms and binds more strongly to this mark [47]. It is evident that HP1β has a more direct functional impact on SUV4-20H2 since loss of HP1β resulted in lower turn-over of the enzyme and reduction in H4K20me3 levels, while loss of HP1a resulted in increased turnover in PCH as opposed to the control group. Furthermore, depletion of HP1β was accompanied by a doubling of cohesin levels and altered localization of CTCF inside the nucleus, with higher levels of this protein observed in major satellites. Experiments of MNase (micrococcal nuclease) activity demonstrated that the absence of HP1β resulted in decompaction of chromatin, but the mechanism did not rely on H4K20me3 since cohesin and CTCF were increased, indicating a possible inhibitory effect of HP1β on cohesin accumulation regardless of H4K20me3 levels. Overexpression of the proteins did not alter SUV4-20H2 dynamics, while modulation of HP1γ levels mainly affected the other two isoforms [47].

Altogether, the three isoforms appear to have distinct effects on SUV4-20H2, with HP1β having a more impactful presence [48]. Further experiments with regard to the levels of HP1 proteins and their association with SUV4-20H1/2 proteins in the formation of PCH in mouse nucleoli demonstrated that nucleolar integrity was not an issue despite the lack of H4K20me3 and the slightly larger nuclei, the bigger chromocenter volumes and the number of chromocenters observed, indicating that, through the different heterochromatic areas, the interactions are diverse and might have distinct roles yet to be defined [44]. 

### 3.4. SUV4-20H2 Participation in Neuronal Differentiation

There is evidence that the KMT5C gene is involved in the process of brain development. Experimental studies on *Xenopus laevis* using morpholino knockdown of SUV4-20 enzymes resulted in problematic eye and melanocyte differentiation, decreased cell proliferation and elevated apoptosis [49]. More precisely, without distinguishing between the two isomorphs, the study demonstrated the decreased levels of H4K20me3, normally found in the first exon of *Oct-25*, which inhibits the action of downstream genes responsible for neural induction. However, upon the knockdown of *Oct-25*, the phenotype was rescued, indicating the necessity of SUV4-20 enzymes for neuroectodermal differentiation [49]. 

On the contrary, the regional Cre-induced depletion of SUV4-20 enzymes did not affect the cellular differentiation of neural progenitor cells, in spite of problems in their cell cycle progression. In greater detail, specific areas of the adult brain of mice were targeted, namely the subgranular zone of dante gyrus and the subventricular zone adjacent to the lateral ventricle. These areas consist of neurogenic niches, high in concentration of progenitor cells, which have high levels of H4K20me3 in cells positive for glial fibrillary acidic protein (*GFAP*), nestin (*NES*) and doublecortin (*DCX*) genes, responsible for life-long neurogenesis [50,51].

Taken altogether, SUV4-20H2 plays an important physiological role in replication, the establishment of heterochromatin, transcriptional regulation and silencing, as well as in the preservation of proper telomere length, affecting important hematological events such as class switch recombination in B cells, the maturation of erythrocytes and the production of ε-globin in embryonic human erythrocytes, as well as the differentiation of embryonic stem cells and brain developmental processes (Table 1). 

## 4. Involvement of SUV4-20H2 in Cancer

**Figure 3:**
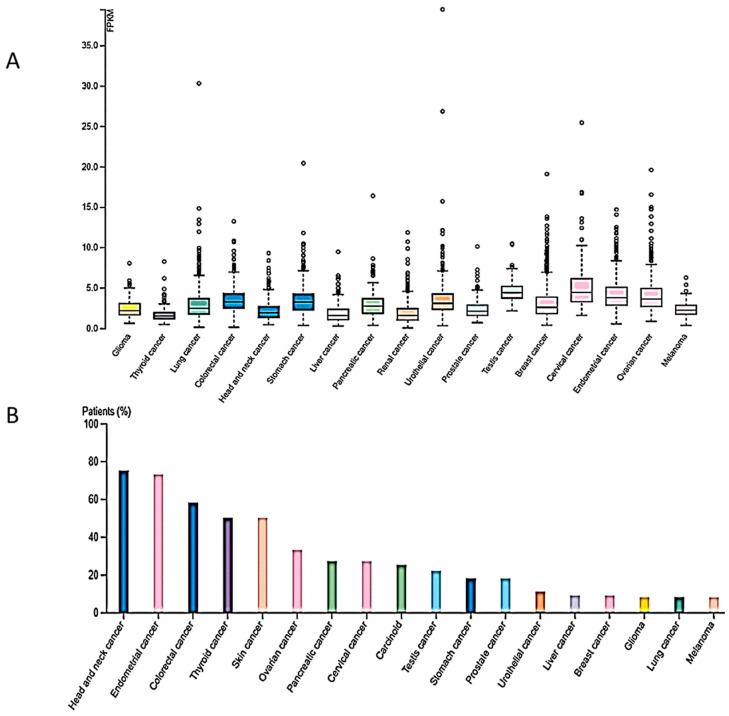
KMT5C RNA-seq expression (TCGA dataset), (**A**) and SUV4-20H2 protein expression (**Β**) in different cancer types (HPA052294, the Human Protein Atlas https://www.proteinatlas.org/ENSG00000133247-KMT5C/pathology, accessed on 22 January 2024).

Given the important role of H4K20me3 in vital cellular functions, it is evident that aberrant expression and activity of SUV4-20H2 may be associated with tumorigenesis. In fact, RNAseq expression analysis from the TCGA dataset revealed the presence of the *KMT5C* gene in several cancer types shown in Figure 3A. Moreover, SUV420H2 protein expression (HPA dataset) was higher in head and neck cancer, endometrial, colorectal, thyroid and skin cancer, as well as in several cases of ovarian, pancreatic, cervical, prostate and stomach cancers, exhibiting moderate to strong cytoplasmic staining along with nuclear positivity in some cases (Figure 3B).

### 4.1. Cancer Types Exhibiting Reduced SUV4-20H2 Expression

Of importance, several cancer types including breast, colon and lung carcinoma exhibit loss of the heterochromatic mark H4K20me3 and reduced SUV4-20H2 expression with disease progression. 

#### 4.1.1. Breast Cancer

More specifically, a study of breast cancer cell lines of different breast cancer subtypes showed that tumor progression was accompanied by significant epigenetic alterations such as loss of DNA methylation, loss of H4K20me3 and hyperacetylation of histone H4 [52]. In particular, MDA-MB-231 and MDA-MB-231(S30) cell lines, characterized by a more invasive and malignant phenotype, bear the aforementioned characteristics in a more exacerbated form as opposed to MCF-7 cells, which are indicative of a more subtle luminal subtype. In addition, lower H4K20me3 levels were accompanied by lower SUV4-20H2 levels as well as altered levels of DNA methyltransferase 1 (DNMT1), methyl-CpG binding protein 2 (MeCP2) and methyl-CpG binding domain protein 2 (MBD2), which are interacting proteins of the H4 trimethylation mark. These findings were recently confirmed in patient samples [53], where higher levels of the enzyme were detected in the neoplastic tissue as opposed to the adjacent healthy cells in the early stages of breast cancer. However, in more advanced stages of the disease, lower levels of SUV4-20H2 were observed compared to healthy tissue. A possible mechanism of SUV4-20H2 action involves transcriptional regulation and, more precisely, repression of genes associated with cell adhesion, such as tensin-3 [54]. It has been shown that SUV4-20H2 establishes its repressive mark upstream of the transcription start site of tensin-3, a focal adhesion protein associated with cancer cell migration, thus inhibiting its transcription and subsequently, the metastasis of breast cancer cells. Τhe tensin family proteins comprise four members, namely tensin 1, tensin 2, tensin 3 and c-ten. All the members of the family bear two important domains in their C-terminal part. The first is the Src homology domain (SH2), which is important for phosphorylation by the Src protein, and the second domain is the phosphotyrosine binding domain (PTB), which mediates interactions with β-integrin. These findings were confirmed through exogenous delivery of the methyltransferase in MDA-MB-231 cells as well as genetic manipulation of MDA-MB-231, resulting in overexpression of SUV4-20H2, while cell invasiveness was substantially diminished. Another mechanism that appears to regulate EMT in breast cancer is the induction of miR-29a by the basic fibroblast growth factor (bFGF) [55]. This protein is mainly produced by microvascular endothelial cells of tumor tissues and promotes cell invasiveness. In response to the production of bFGF, miR-29a is transcribed and binds to the 3′UTR of SUV4-20H2 mRNA, thus blocking its production. As a result, suppression of connective tissue growth factor (CTGF) and growth response protein-1 (EGR-1) were attenuated, leading to EMT progress of breast cancer cells. It is of note that miR-29a was detected to be elevated in breast cancer stem cells and involved in tumor formation, metastasis and drug resistance, indicating a possible therapeutic target.

#### 4.1.2. Colon Cancer

Studies addressing the role of SUV4-20H2 in colon cancer (CC) have shown that murine and patient-derived colorectal cancer organoids exhibit low levels of H4K20me3 in right-sided CC (RCC) patients and high chromatin accessibility [56]. However, it was reported that the methyltransferase levels did not differ between right and left CC patients, which was attributed to lower enzymatic activity. The higher chromatin accessibility was studied through MNase assay and the phenotype was rescued through methylstat, introduced to mice bearing tumors made from RCC patient-derived organoids. In addition, murine colon-derived organoids bearing mutations associated with RCC, namely Tgrbr2-deficient and K-ras G12D, exhibited decreased H4K20me3 and SUV4-20H2 levels, along with loosely packed chromatin. The compaction was ameliorated by applying methylstat, which resulted in larger tumors too. In a similar murine organoid of RCC, knockdown of SUV4-20H2 was shown to induce more aggressive orthotropic tumors, lower chromatin compaction and enrichment of genes targeted by the Wnt pathway such as leucine-rich repeat-containing G-protein coupled receptor 5 (*Lgr5*), SRY-box transcription factor 2 and 9 (*Sox2*, *Sox9*) and prominin-1 (CD133). It is worth highlighting that these genes are localized in LAD (lamina-associated domain) areas in mouse embryonic fibroblasts, leading to the hypothesis that the decrease in SUV4-20H2 levels allows for the dissociation of LAD areas from the nuclear lamina, thus attenuating gene repression. Chromatin immunoprecipitation experiments targeting the promoter of *Lgr5* confirmed this assumption and the interplay of regulating gene expression through chromatin compaction and association with the nuclear membrane. 

#### 4.1.3. Lung Cancer

A correlation between SUV4-20H2 levels and tumor development has been suggested in an immunohistochemical study of lung cancer patients, where H4K20me3 changes were observed in squamous cell carcinoma. H4K20me3 staining in early precursor lesions decreased significantly with disease progression and was associated with reduced SUV4-20H2 expression [57].

### 4.2. Cancer Types Exhibiting Increased SUV4-20H2 Expression

#### 4.2.1. Pancreatic Cancer

In pancreatic cancer, the role of SUV4-20H2 appears to be different and related to the regulation of genes responsible for mesenchymal phenotype, which is associated with higher invasiveness and drug resistance. While screening pancreatic ductal adenocarcinoma (PDAC) human samples, it was shown that the SUV4-20H2 reported reduced levels, contributing to the establishment of the epithelial state, as demonstrated through E-cadherin and epithelial cell adhesion molecule (EPCAM). On the contrary, higher levels of SUV4-20H2 establish the H4K20me3 repressive mark that blocks the expression of epithelial transcriptional marks and allows for the mesenchymal state to be developed, as reported through vimentin levels [58]. Furthermore, it was shown through bioinformatic analysis that knockdown of SUV4-20H2 results in the activation of genes involved in cell adhesion, cytoskeleton and extracellular matrix as well as markers related to epithelial state. Functional studies demonstrated that the knockdown of the methyltransferase is related to reduced migration and invasion potential. Moreover, higher sensitivity to drugs as well as lower levels of CD24 and CD44 were reported, indicating a reduction in cancer stem cells, which bear the ability to form tumors of pancreatic origin in xenografts [58]. It is worth pointing out that SUV4-20H2 exerts the aforementioned effects through transcriptional regulation of MET-associated transcriptional factors, namely forkhead box A1 (FOXA.1) and Ovo-like transcriptional repressor 1 and 2 (OVOL1/2) in pancreatic cancer, which in turn have the ability to activate different downstream signaling pathways. It is of interest that the epigenetic mark H4K20me3, established by SUV420H2, was differentially placed on gene loci associated with transcription factors implicated in mesenchymal and epithelial states. The levels of the mark dropped in the case of FOXA.1 and OVOL1/2, but no changes were reported for Cadherin 2 (CDH1) and EPCAM, suggesting the indirect regulatory effect of SUV4-20H2 on epithelial state-related genes. Furthermore, in pancreatic cell lines with a more epithelial-like phenotype, overexpression of SUV4-20H2 demonstrated the repressed levels of Cadherin 2 (CDH2) and EPCAM as well as increased levels of vimentin, implicating the enzyme in the regulation of the epithelial to mesenchymal transition. Finally, immunofluorescent staining of patient-derived tissues showed a correlation of SUV420H2 expression with the mesenchymal state and disease progression [58]. 

#### 4.2.2. Renal Cell Carcinoma 

In renal cell carcinoma, SUV4-20H2 was shown to epigenetically regulate cell proliferation through targeting of dehydrogenase/reductase 2 (DHRS2) [59]. Using the A498 clear cell renal cancer cell line, representative of the majority of kidney cancers, it was demonstrated that silencing of the methyltransferase suppressed growth and induced cell apoptosis through attenuation of H4K20me3 levels on the promoter of *DHRS2*. It is of note that simultaneous silencing of *KMT5C* and *DHRS2* rescued the phenotype, while the use of SUV4-20 family protein inhibitor A-196 resulted in cell apoptosis by increasing the levels of DHRS2. Furthermore, the research data highlight the higher levels of SUV4-20H2 in renal cell carcinoma patients (Table 2) and their association with poor prognosis [59].

#### 4.2.3. Hepatocellular Carcinoma

The implication of SUV4-20H2 in hepatocellular cancer was revealed in the F344 rat model of endogenous hepatocarcinogenesis [60] after treatment with the methyl-deficient diet (MDD), which showed a gradual loss of DNA methylation on repetitive elements, followed by the loss of H4K20me3 and the gradual decline of SUV4-20H2 levels with diseases progression. It was evident that hepatocellular carcinogenesis is associated with the gradual loss of histone H4 trimethylation due to lower enzyme activity (Table 2). Recently, it was shown that higher levels of H4K20me3 were associated with worse prognosis for hepatocellular carcinoma patients and suggested the reactive oxygen species (ROS) as one of the regulatory pathways for the induction of the epigenetic mark [61]. In HepG2 and Huh7 cell lines, the study demonstrated that during oxidative stress induced by the presence of H_2_O_2_, the epithelial marker E-cadherin is downregulated, whereas a-SMA and MMP-9 mesenchymal markers are increased. The repressive or activating pathways are initiated by ROS, but the underlying mechanism is not specified. In any case, cancer aggressiveness was substantially increased through the activation of EMT. These findings were further confirmed after treatment with the SUV4-20H2 inhibitor A-196, which resulted in decreased epigenetic mark levels and the EMT phenotype. 

Table 2 summarizes the studies indicating an implication of SUV4-20H2 in different cancer types.

## 5. Targeting Options of SUV4-20H2

Our understanding of the epigenetic interplay in pathological conditions, including cancer, and the complexity lying beneath the cross-talk of a diverse set of epigenetic marks has been further advanced through the development of different compounds with inhibitory effects [62,63,64]. It is of note that HMTs bear two structurally distinct and important sites that have been targeted for inhibition. The first site allows for the docking of S-adenosyl-L-methionine (SAM), which is the cofactor of the enzymatic methylation process of lysine residues. The second site located on HMTs is the docking site of histone tails, where the methylation mark will appear by the end of the process [65]. Inhibition can be achieved by targeting one of these two sites separately by using either mimicking compounds of the cofactor or blocking the pocket where the histone tail binds. Furthermore, the development of high-throughput screening assays has enabled the identification of a wide variety of inhibitors acting through different mechanisms such as allosteric negative regulation and noncovalent or covalent molecules. It is important to note that a well-thought-out pipeline of refinement is gradually developing with an interplay between structural biology and computational chemistry. Experimental studies that shed light on the crystal structures of interacting molecules and docking analyses, as well as assays that allow for the evaluation of molecule’s potency, selectivity, binding affinity and target engagement, have allowed the careful adjustment of initially discovered compounds with inhibitory effects and the amelioration of their action [66]. 

An example of the combination of these technologies is A-196, a compound that can inhibit both SUV4-20H1 and SUV4-20H2 [67,68]. A-196 has the ability to bind the active site where the enzymatic reaction is taking place without affecting the binding site of the cofactor. As a result, H4 cannot properly enter the pocket, and therefore, di- or trimethylation, depending on the enzyme, cannot occur. For this interaction to take place, two amino acids, namely isoleucine 231 and tryptophane 264, need to change their initial place so as to create a hydrophobic area. Due to the replacement of these two amino acids, the bigger part of the compound, containing two negatively charged chloride atoms, can enter the active site. For the rest of the molecule, especially the cyclopentene, to enter the pocket, two more amino acids need to alter their position, Met-253 and Phe-311. It is important to note that SUV4-20H1 and SUV4-20H2 have nearly identical homologies in their active site with the exception of proline instead of serine in SUV4-20H2. This difference was pointed out as a possible reason for the lower inhibition detected with regard to SUV4-20H2 as opposed to SUV4-20H1 in studies performed in two distinct cell lines, U2OS osteosarcoma and LnCaP prostate cancer. 

During monitoring of the compound’s efficiency through the decreased di- and trimethylation of H4K20 and the increased levels of monomethylated H4K20 throughout the cell cycle, additional effects were detected. Firstly, A-196 has the ability to block nonhomologous end-joining (NHEJ) repair of the double-stranded DNA breaks. The mechanism for this process includes the binding of 53BPI foci and the pharmacological inhibition of the enzymes, resulting in the absence of di- and trimethylated H4K20, indicating the importance of these marks for NHEJ. Furthermore, given that NHEJ is a very important step for class switch recombination taking place in immune cells, primary murine spleen cells were used to prove the effect of a dysfunctional NHEJ system due to the lack of H4K20me2/3 after inhibition of SUV4-20H1/2. Secondly, the compound appears to have no effect on cell viability or cause any form of resistance. 

A novel drug that has recently been proposed to work against histone-methylating enzymes is vorinostat. The drug has been initially proposed to work against histone deacetylases (HDAC) I, II and IV and as such has been used for patients with advanced or even chemotherapy-resistant T cell lymphoma [64,69]. However, a recent study using vorinostat on the HeLa TI cell line demonstrated that the drug might be able to target histone methylases as well. This particular cell line has a *GFP* reporter gene which is epigenetically silenced by a combination of 15 different proteins, including some histone methyltransferases and HDAC1. Given that these 15 factors can interact either directly or indirectly and through various signal pathways, different inhibitors can elucidate their interactions as well as the contribution of each factor to the final silencing of *GFP*. The study showed that the percentage of *GFP*+ cells was 2.1 times higher during HDAC1 knockout and vorinostat exposure as opposed to HDAC1 knockout or drug exposure alone. Upon SUV4-20H1 knockout, an 8.8-fold increase in *GFP*+ cells was observed as opposed to the control. Moreover, a combination of SUV4-20H1 knockout and vorinostat resulted in 63.2% *GFP*+ cells, indicating the clear impact the compound has on the enzyme. Given that SUV4-20H1 and SUV4-20H2 exhibit great structural homology, one could expect that vorinostat might have the same impact on SUV420H2 knockout cells. To this end, the levels of SUV4-20H2 were decreased after treatment of the HeLa TI cell line with vorinostat, the decrease being 1.3-fold for SUV420H1 and 1.5-fold for SUV4-20H2 [70].

## 6. Clinical Trials Based on Histone Methyltransferase Activity

Taking into account the increasing body of evidence that implicates histone methylation in cancer onset, development, progression and metastasis, different compounds with inhibitory effects are being used in clinical trials aiming to identify writers, erasers or even readers of epigenetic marks. Different strategies are deployed in their development that include targeting active sites and mimicking SAM (s-adenosyl-L-homocysteine), including carbamate or urea/benzimidazole-containing compounds [71]. Several clinical trials targeting histone methyltransferases such as KMT2A, DOT1L and EZH2, which are writers of epigenetic marks such as SUV4-20H2, have given promising results and are described in this section. To date, SUV4-20H2 has not been targeted for cancer treatment, probably because of the small body of evidence available with regard to its role in the initiation, development and progression of cancer.

### 6.1. Targeting of KMT2A and DOT1L to Combat Different Forms of Myeloid Leukemia

KMT2A also known as lysine methyltransferase 2A is responsible for the establishment of mono- and demethylation of H3K4 via a nonprogressive mechanism [72]. This epigenetic mark has gene-activating effects and is particularly important for early development and hematopoiesis [73,74]. Normally the *KMT2A* gene is localized in chromosome 11q23. However, in acute myeloid leukemia (AML), translocation events take place that lead to the production of a variety of fusion proteins and the loss of the SET domain required for the catalytic activity of the methyltransferase. As a result, the fusion proteins interact with *DOT1L* (disruptor of telomeric silencing-1), which is the only gene responsible for the mono-, di- and trimethylation of H3K79, an activating epigenetic mark that regulates the expression of proleukemogenic genes such as *HOXA9* (homeobox A9) and *MEIS1* (homeobox protein Meis 1). 

Two different clinical trials, (NCT01130506) [75] and (NCT04065399) [76], have been designed to explore targeting of different stages of this pathway. In the first clinical trial, *KMT2A*, which is epigenetically silenced in partial tandem duplication, was re-expressed with the use of a regime consisting of decitabine, vorinostat and cytarabine in a group of 17 adults with relapsed or refractory AML. The treatment regime consisted of intravenous infusion of decitabine at a dose of 20 mg/m^2^/day on days 1–10, vorinostat was given orally at a dose of 400 mg/day on days 5–10 and cytarabine was infused intravenously twice a day at days 12, 14 and 16. Among the three drugs, only cytarabine dosage was altered and gradually escalated from 1.5 to 3 g/m^2^. The overall response rate for this study was 35%, with 6 out of 17 patients presenting complete recovery with a median number of two prior therapies at a range of 1 to 3. However, all patients with complete recovery apart from one who relapsed. 

In the second clinical trial (NCT04065399), a menin inhibitor was prescribed orally in a continuous cycle of 28 days in two parallel dose escalation cohorts, one of which received a CYP3A4 inhibitor. The group consisted of heavily pretreated individuals suffering from relapsed or refractory acute leukemia with a median of four previous lines of therapy, and 44% of patients had an allogeneic stem cell transplant previous to participation in the trial. In this study, patients with mutations both in *KMT2A* and *NPM1* (nucleophosmin 1) were evaluated, given that both the rearranged form of *KMT2A* and the wt (present when NPM1 is mutated) interact with menin, resulting in the expression of *HOX* genes. The menin inhibitor used was revumenib, which resulted in an overall response rate of 53% (32/60 patients) and a complete remission or remission with partial hematologic recovery reaching a 30% percentage. 

DOT1L, a methyltransferase that regulates the methylation levels of a very particular epigenetic mark, H3K79me(1-3) [77], consists of a valuable target for the development of therapies for AML. In a phase 1 clinical trial (NCT01684150), 51 adult patients with advanced acute leukemia, mainly mixed lineage, were prescribed pinometostat (EPZ-5676) [78]. This inhibitor was designed by using the SAM cofactor that provides the methyl group as a template and subsequent treatments to ameliorate pharmacokinetics. The patients were divided into two cohorts, one for dose escalation and one as an expansion cohort. Intravenous infusion of pinometostat at two different concentrations was used, 54 mg/m^2^ and 94 mg/m^2^ per day for 28 day cycles. Important observations during the study include the reduction in H3K79me2 both globally and locally at specific genes implicated in mixed myeloid leukemia such as *HOXA9* and *MEIS1*. However, the reduction levels did not correlate with clinical outcomes, while the degree of reduction was not the same across patients with similar genetic backgrounds. Only two patients achieved complete remission, both bearing similar translocations (11:19), having received extensive treatment before participation in the trial and, unfortunately, relapsing at different time points, while all patients gradually developed at least one adverse effect due to drug use and gradually dropped out of the trial. It was suggested that remission might require constant inhibition of DOT1L and that the presence of different fusion proteins may lead to different sensitivity to DOT1L inhibition. 

### 6.2. Targeting of EZH2 in Various Cancers 

The EZH2 methyltransferase belongs to the SET1 family and is a part of the polycomb repressive complex (PRC2) that confers to its enzymatic activity [79]. As a writer of epigenetic marks, it is responsible for the sequential trimethylation of H3K27, a silencing mark. Given the role of PRC2 in gene silencing, it is no wonder that deregulation of EZH2 levels is associated with cancer development and progression, thus rendering the protein an ideal target for drug treatment. 

In a phase II clinical trial (NCT03456726), a group of 20 Japanese patients suffering from relapsed or refractory B cell non-Hodgkin lymphoma with mutations in EZH2 was administered 800mg of tamezostat twice a day until disease progression or unacceptable toxicity [80]. The molecule is a selective and reversible small inhibitor for EZH2. The patients were divided into two cohorts based on the lymphoma type. In cohort 1, patients (n = 17) with follicular lymphoma demonstrated an objective response rate of 76.5% with 35.3% amounting to complete recovery and 41.2% presenting only partial response. In cohort 2, patients (n = 3) suffering from diffuse B cell lymphoma were included, and they developed only partial response. For the follicular cohort, progression-free survival at 12 months was 94.1%, and at 15 months, it was 73.2%. 

In a phase II clinical trial that took place in 16 hospitals across France, the UK and the USA, tamezostat was employed against malignant pleural mesothelioma, which is an aggressive form of cancer that develops on the surface of lung mesothelium [81]. In this study, 74 patients, 13 in the first part of the trial, aiming at the determination of pharmacokinetics, metabolism and the correct dosage of patients, and 61 in the second part of the trial, aiming to study response to treatment, were enrolled. Patients were older than 18, had received at least one form of treatment and 99% of them had BAP1 (BRCA1-associated protein 1) inactivated tumors. No patient presented a complete response, but two developed a partial response to treatment, which lasted for different time intervals (18 and 42 weeks). Another clinical trial (NCT03603951) targeting EZH2 was the first human study investigating the safety, pharmacokinetics, pharmacodynamics and preliminary clinical outcomes of SHR2554, an oral selective inhibitor of EZH2 [82]. The inhibitor was prescribed to patients suffering from mature lymphoid neoplasms, such as B and T cell lymphomas and classical Hodgkin lymphoma, conducted in China. A total of 113 patients participated in the phase I clinical trial, among whom 107 had adequate response to drug treatment and were included in the downstream analysis and 46 out of them, 43%, developed an overall response, resulting in the conclusion that SHR2554 has an acceptable safety profile and anticancer activity. In Table 3, the main parameters and findings of clinical trials targeting histone methyltransferases are summarized.

## 7. Conclusions and Future Perspectives

Recent experimental data demonstrate that homeostasis maintenance and normal physiology depend heavily on proper gene expression regulation, and deviations from these states result in the development of cancer. Many regulatory mechanisms are taking place, including an extensive interplay of epigenetic modifications that enable cancer development and progression. 

SUV4-20H2 establishes the suppressing epigenetic mark H4K20me3 and has an important role in normal physiology by participating in DNA replication, regulation of embryonic development, promotion of embryonic stem cell differentiation and suppression of lineage-specific genes in ES cells. The methyltransferase role in recombination is also highly significant, as it allows for the maintenance of telomeric length as well as the class switch recombination necessary for the production of antibodies and maturation of B cells. However, most studies indicate the pivotal role of SUV4-20H2 in transcription through chromatin accessibility and RNA pol II regulation, responsible for RNA pol II promoter proximal pausing and the establishment and maintenance of heterochromatin, a process necessary for erythrocyte maturation and neuroectodermal differentiation. 

Given the core processes in which the methyltransferase is involved, it can be expected that the deregulation of its levels results in carcinogenesis. Several cancers such as breast, colon and lung tumors are characterized by genome-wide loss of SUV4-20H2 and of the heterochromatic H4K20me3 mark possibly involved in increased telomere elongation, increasing cell growth rate and tumorigenicity, further correlating with poor prognosis. However, at the same time, increased SUV4-20H2 expression has been observed in certain tumors, possibly attributed to gene amplification and acting as a switch of the epithelial or mesenchymal state, demonstrating a heterogeneous regulation motif in association with the cancer tissue in question. In pancreatic cancer, high levels of the methyltransferase regulate ΕΜΤ-associated transcriptional factors and exhibit a prognostic potential, which has been correlated with worse survival, whereas in breast cancer and RCC, SUV4-20H2 inactivation or depletion results in the establishment of an epithelial state. 

It is important to note that continuous research focused on SUV4-20H2’s functional role in cancer is required, along with a more in-depth investigation of the regulatory mechanisms underlying SUV4-20H2 expression and activity. To this end, specific antibodies for SUV4-20H2 are in high demand for use in ChIP-seq methodology as well as immunofluorescence and proximity ligation assays to detect additional interacting proteins as well as signals that stimulate SUV4-20H2 functions. Moreover, specific SUV4-20H2 inhibitors need to be synthesized for the cancer types that exhibit high levels and activity of this methyltransferase to determine preclinical effects in experimental models and allow future administration in clinical trials.

## Figures and Tables

**Table 1:** Main physiological functions of SUV4-20H2 in different cell types.

Molecular Process	Role of SUV4-20H2	Cell Type	Reference
Recombination	Maintenance of telomeric length homeostasis	Μouse embryonic fibroblasts, murine embryonic stem cells	[43]
	Immunoglobulin class switch recombination	Μature B cells	[40,41]
Transcriptional regulation	RNA pol II promoter proximal pausing	Μature erythrocytes	[40,41,42]
	Direct regulation of *Oct-25* gene during neuroectodermal differentiation	Μelanocytes and eye cells from *Xenopus laevis*, neural progenitor cells of murine brains from the subgranular zone of dante gyrus and the subventricular zone adjacent to the lateral ventricle	[49,50,51]
	Repression of lineage-specific genes in ES cells, embryonic stem cell differentiation	Εmbryonic stem cells	[38,39]
	Control of chromatin architecture, namely the establishment of heterochromatin	Ιn diverse cell types, very important for red cell maturation	[44,46,47,48]
DNA replication	Recruitment of origin of recognition complex (ORC) to replication origin sites	Mouse embryonic fibroblasts	[35]
	Assistance in the correct replication time in heterochromatic regions	-	[37]

**Table 2:** SUV4-20H2 implication in different cancer types.

Cancer Type	SUV420H2 Expression	H4K20me3 Expression	Main Effects	Reference
Breast	High levels in low-grade tumors Low levels in high-grade tumors	Low	Repression of genes responsible for cell adhesion, tensin-3 blocked by miR-29a	[50,51]
Colon	Low levels	Low	Enrichment of genes implicated in Wnt pathway and dissociation of LAD areas in mouse embryonic fibroblasts, attenuating gene repression	[52]
Lung	Low levels	Low	Loss of H4K20me3 correlates with worse survival in stage I adenocarcinoma patients	[53]
Pancreatic	High levels	High	Regulation of MET-associated transcriptional factors, FOXA1 and OVOL 1/2, regulation of epithelial or mesenchymal state	[54]
Renal cell carcinoma	High levels	High	Maintenance of H4K20me3 levels on *DHRS2* promoter to maintain growth and avoid apoptosis	[55]
Hepatocellular	High levels in early stages, which drop with disease progression	Low	Reduction in epithelial marker E-cadherin and increase in mesenchymal markers SMA and MMP-9	[57]

**Table 3:** Clinical trials targeting histone methyltransferases.

Clinical Trial Identifier	Targeted HMT	Drug	Diagnosis/Cancer Type	Patients (Number, Genetic Characteristics, Previous Treatments)	Results	Reference
NCT1130506	KMT2A (lysine methyltransferase 2A)	Dacitabine (intravenously at 20 mg/m^2^/day on days 1–10), vorinostat (oral administration at 400 mg/day on days 5–10), cytarabine (intravenous infusion twice a day on days 12, 14 and 16 with gradual increase in dosage from 1.5 g to 3 g/m^2^.	Acute myeloid leukemia (AML) relapsed or refractory	17 adults with rearrangements in *KMT2A* gene and a median of 2 prior treatments (range 1–3)	35% overall response with 6/17 presenting complete response. 5/6 relapsed	[75]
NCT04065399	KMT2A	Revumenib (oral administration in two parallel dose escalation cohorts, one receiving a CYP3A4 inhibitor and the other not)	Acute myeloid leukemia (AML) relapsed or refractory	60 patients with mutations in *KMT2A* and *NPM1* gene all heavily medicated with a median of 4 previous treatments, 44% received allogeneic stem cell transplant before participation	53% overall response with 32/60 presenting complete response and complete remission or remission with hematologic response reaching 30%	[76]
NCT01684150	DOT1L (disruptor of telomeric silencing 1)	Pinometostat (EPZ-5676) (intravenous administration at two different doses of 54 mg/m^2^ and 94 mg/m^2^ per day)	Advanced acute leukemia, mainly mixed lineage	51 patients enrolled in two cohorts (n = 26 for the 6 dose escalation phase and n = 25 for the expansion cohort), translocations for the MLL gene (*KMT2A*) were present in the patients but not all of them had the same or similar translocation	Only 2 patients achieved complete remission bearing translocations (11:19) but the duration of remission was different and both relapsed eventually	[78]
NCT03456726	EZH2 (enhancer of zeste homologue 2)	Tamezostat (administration twice a day at 800 mg)	B cell non-Hodgkin lymphoma	20 patients bearing mutations in *EZH2*, divided in two cohorts (n = 17 with follicular lymphoma and n = 3 with diffuse large B cell lymphoma)	Cohort 1: objective response rate 76.5% with 35.5% amounting to complete recovery and 41.2% to partial response, with progression without events at 12 months being 94.1% and at 15 months, 73.2%; Cohort 2: only partial response	[80]
NCT02860286	EZH2	Tamezostat	Malignant pleural mesothelioma	74 patients (13 for the first part of the trial and 61 for the second), at least one form of treatment prior to participation, 99% had BAP1 inactivated tumors	No patient presented complete response to treatment but two presented partial response that lasted different time intervals (18 and 42 weeks)	[81]
NCT03603951	EZH2	SHR2554	Mature lymphoid neoplasms, B and T cell lymphomas, classical Hodgkin lymphoma	113 patients with 107 used for analysis	46/107, 43%, developed an overall response to treatment	[82]

## Data Availability

Not applicable.
